# Combined analysis of transcriptome and metabolome reveals that sugar, lipid, and phenylpropane metabolism are essential for male fertility in temperature-induced male sterile rice

**DOI:** 10.3389/fpls.2022.945105

**Published:** 2022-07-28

**Authors:** Yujun Sun, Ming Fu, Yina Ang, Lan Zhu, Linan Wei, Ying He, Hanlai Zeng

**Affiliations:** ^1^MOA Key Laboratory of Crop Ecophysiology and Farming System in the Middle Reaches of the Yangtze River, College of Plant Science and Technology, Huazhong Agricultural University, Wuhan, China; ^2^Center of Crop Nanobiotechnology, Huazhong Agricultural University, Wuhan, China

**Keywords:** temperature changes, PTGMS rice, male sterility, pollen wall, metabolomic analysis

## Abstract

Photoperiod- and thermosensitive genic male sterility (PTGMS) rice is a vital germplasm resource consisting of two-line hybrid rice in which light and temperature strictly control their fertility changes. Variable environmental conditions present huge risks to the two-lines hybrid seed production. Explaining the regulatory mechanism of male fertility in rice PTGMS lines is an essential prerequisite to ensuring food security production. A group of near-isogenic lines (NILs) of a rice PTGMS line unique to this research group was used for this study. These lines have the same genetic background and regulate male fertility by responding to different temperature changes. Transcriptomic analysis revealed that 315 upregulated genes and 391 regulated genes regulated male fertility in response to temperature changes, and differentially expressed genes (DEGs) were mainly characterized in enrichment analysis as having roles in the metabolic pathways of sugar, lipid and phenylpropanoid. Electron microscopy analysis revealed that a lack of starch accumulation in sterile pollen grains induced by high temperature, with an abnormal exine development and a lack of inner pollen grains. Defective processes for sporopollenin synthesis, sporopollenin transport and pollen wall formation in sterile anthers were verified using qPCR. Targeted metabolomics analysis revealed that most lipids (phospholipids, sphingolipids and fatty acids) and flavonoids (flavones and flavanones) were upregulated in fertile anthers and involved in pollen wall development and male fertility formation, while lignin G units and C-type lignin were the major contributors to pollen wall development. The coding genes for trehalose 6-phosphate phosphatase, beta-1,3-glucanase, phospholipase D and 4-coumarate-CoA ligase are considered essential regulators in the process of male fertility formation. In conclusion, our results indicated that the expression of critical genes and accumulation of metabolites in the metabolism of sugar, lipid, and phenylpropanoid are essential for male fertility formation. The results provide new insights for addressing the negative effects of environmental variation on two-line hybrid rice production.

## Introduction

As an essential food crop, rice is a staple food for approximately half the world’s population, and in China, this population is more than 60%. (Khush, 2005). China is the largest producer and consumer of rice in the world, and harnessing heterosis is one of the primary ways to increase rice production. Hybrid rice breeding started in China in the 1970s, and the yield of hybrid rice has risen by more than 20% compared with that of conventional rice (Cheng et al., 2007; Ma and Yuan, 2015). Currently, hybrid rice breeding is produced through “three-line systems” or “two-line systems” based on male sterile lines for mass reproduction and production. Two-line hybrid rice uses photoperiod- and thermosensitive genic male sterility (PTGMS) lines to produce hybrid seeds, and its fertility is strictly controlled by light and temperature. The production of PTGMS hybrid seeds requires stringent environmental conditions, The variable environmental conditions present huge risks in the small window of critical temperature for fertility transformation (CTFT) in PTGMS seeds and hybrid breeding (Mou, 2016). In addition, the CTFT of rice PTGMS lines will increase with the breeding process. The PTGMS individuals must be reisolated repeatedly during hybrid seed production to reduce the risk of seed production from an increase in fertile temperature (Chen et al., 2011; Liao et al., 2021). Therefore, understanding the molecular mechanisms that respond to temperature changes and regulate fertility is the essential way, for avoiding the negative effects of environmental changes on hybrid breeding.

Currently, at least 13 PTGMS loci have been identified in rice (Fan and Zhang, 2018). For instance, loci *PMS1* and *PMS2* were identified in the PGMS line of NK58S progeny, while another locus *PMS3* was mapped on chromosome 12. Further studies have revealed that the original mutation in *PMS3* caused the change from NK58 to NK58S (Mei et al., 1999; Li et al., 2001). In the TGMS line Peiai64S, the sterility gene *p/tms12-1* has been cloned. *p/tms12-1* is identical to the locus *pms3* cloned in NK58S, and a loss RNA function due to a C-G base substitution in *p/tms12-1* results in male sterility in Peiai64S (Ding et al., 2012). Although several effector genes in rice PTGMS lines have been more deeply investigated, the specific genetic mechanisms regulating male fertility in response to temperature changes are still unclear.

With the development of whole-genome sequencing technology and metabolomics, an increasing number of regulators (genes, miRNAs, circRNAs, and lncRNAs) and metabolites have been confirmed to be involved in the regulation of male fertility in rice PTGMS lines (Ding et al., 2012; Wang et al., 2019, 2021; Zhu et al., 2020; Sun et al., 2021). For instance, lncRNAs act as an endogenous target mimics to regulate the gene expression levels of *GRF* and *SPL* by competing with miR396 and miR156 to involve in the fertility transformation process of rice PTGMS lines (Wang et al., 2021). Our previous research also found that the miR156-*SPL* module may regulate male fertility in response to temperature changes, and *OsSPL* regulates the activity of APX, thereby regulating rice male fertility by controlling the programmed cell death (PCD) process in the tapetum. A recent study found that the GA negative regulator DELLA/SLR1 regulates premature PCD in the tapetum and affects the development process of the anthers (Jin et al., 2022). Delayed degradation of the tapetum and abnormal development of the exine will lead to pollen abortion in some mutants, while the development of male gametophytes mainly occurs by integrating carbohydrate and fatty acid metabolism (Zhang et al., 2021). Therefore, the timely PCD in the tapetum and an intact pollen wall are crucial for the formation of male fertility, while the accumulation of carbohydrate and lipid metabolites determines the male fertility in rice (Wan et al., 2020; Liu et al., 2021).

The mature pollen of rice is surrounded by a pollen wall with a multi-layered shell. The pollen wall protects against microbial invasion and abiotic stresses during pollen development, and its integrity is essential for maintaining normal pollen function (Ariizumi and Toriyama, 2011; Jiang et al., 2013). The mature pollen wall consists of exine and intine, with the exine consisting of an inner nexine and the outer sexine connected by bacula to constitute an I-shaped structure (Shi et al., 2015). Synthesis of the pollen wall starts at the meiotic stage, the degradation of the tapetum produces callose to form the primexine as the primary structure of the exine, and then the sporopollenin accumulates to create the complete exine structure (Wilson and Zhang, 2009; Wallace et al., 2011). Sporopollenin consists mainly of fatty acids and phenylpropanoid metabolites that serve to coat and protect pollen grains (Wallace et al., 2011). Therefore, sporopollenin’s normal synthesis and transport are essential for forming fertile pollen. Some studies have found that the sporopollenin biosynthesis gene mutants (*CYP703A3*, *CYP704B2*, and *DPW*) exhibited defective sporopollenin biosynthesis and abnormal pollen wall structure in rice (Li et al., 2010; Shi et al., 2011; Yang et al., 2014). ABC family proteins are important lipid transport proteins in eukaryotes, and mutations in the *OsABCG26* gene cause pollen sterility. *OsC6* is responsible for transporting lipidic precursors to form the anther epidermis and the exine, and knockdown of the *OsC6* gene leads to aborted pollen exine (Zhang et al., 2010; Luo et al., 2020). This suggested that the biosynthesis of sporopollenin and the transport of lipids in the anther are crucial for forming pollen walls and pollen grains.

Pollen intine is the innermost pollen wall under the exine, it consisting mainly of substances such as cellulose, hemicellulose and pectin. It is widely believed that intine is inseparable from the formation of microspores (Ariizumi and Toriyama, 2011). At later stages of pollen development, pollen begins to accumulate starch, which is essential for pollen maturation. In some studies, mutants of the Golgi-localized glycosyltransferase (*OsGT1*), the arabinosy l kinase-like protein (*CAP1*) and the UDP-arabinopyranose mutase (*OsUAM3*) have been shown to have a disrupted inner structure and male sterility in rice, which was demonstrated to be essential for inner development (Moon et al., 2012; Ueda et al., 2013; Sumiyoshi et al., 2014). In addition, silencing *OsUGP2*, a member of the rice UGP enzyme family, causes impaired starch accumulation in pollen grains and leads to male sterility. Similarly, deficiencies in *OsAGPL4, OspPGM*, and *OsHXK5* can reduce the synthesis of pollen starch, leading to male sterility (Mu et al., 2009; Lee et al., 2016, 2019). Therefore, sugar metabolism and related genes are essential for pollen inner wall development and starch accumulation in pollen grains. In this study, we used unique near-isogenic lines (NILs) with different CTFTs to find DEGs that respond to temperature changes and are involved in fertility regulation; combined with comparative transcriptome and targeted metabolomics analysis, we elucidated the potential roles of critical genes and metabolites in the metabolic pathway on pollen wall development and male fertility formation, which provides insights for in-depth elucidation of the male fertility mechanism and environmental adaptation of rice PTGMS lines.

## Materials and methods

### Temperature treatment for experimental materials

We selected a group of NILs PA2364S, PA2864S, and PA2864F, which are unique materials obtained by our research laboratory from Peiai64S (PA64S) after multigenerational selection and breeding (Zeng and Zhang, 2001). PA2364S (abbreviated as P23) is a rice PTGMS with CTFT of 23°C and shows male fertility at long daylight (LD) and average temperature below 23°C; conversely, it showed male sterility at an average temperature above 23°C. The photo-thermal response of PA2864S is consistent with that of PA2364S, which has a CTFT of 28°C. PA2864F (abbreviated as P28F) is conventional rice and shows high fertility and seed setting rates under different temperature treatments. This experiment was conducted during 2020–2021 at Huazhong Agricultural University (30.28°N, 114.20°E). The seeds were sown in May. The better growing seedlings were selected and transplanted to enamel pots in June. In each pot, we transplanted three seedlings under the same growing conditions. When 50% of the young rice spikelets developed to the secondary stalk and spikelet primordium differentiation stage, uniformly growing plants were selected and moved into plant growth chambers at an average temperatures of 23°C and 30°C, respectively. The light conditions set in the growth chamber was 300 μmol/(m^2-s^) and the relative humidity was 80% (Supplementary Figure 1).

### Anther phenotype and fertility statistics of rice

According to our previous research methods, representative mature rice plants and spikelets were selected for observation and photography using a stereomicroscope to develop I_2_-KI staining and seed setting rate statistics of rice (He et al., 2021a; Sun et al., 2022). Briefly, we picked the top florets that had initiated spikelets, pinched out the anthers with forceps, squeezed out the pollen grains into 1% I_2_-KI solution, and observed and counted them under a microscope. When the rice was mature, two spikelets were randomly cut from the same plant and replicated in 60 groups to calculate the seed setting rate. The sample size for the pollen fertility and seed setting rate was *N* > 120, and significant differences were analyzed based on a *t*-test.

### RNA extraction and transcriptome sequencing

The young spikelets at different developmental stages were sampled and divided into 14 anther developmental stages according to the correspondence between anther length and anther developmental stages (Zhang et al., 2011). The 9th stage anther of P23, P28, and P28F were selected to extract total RNA for the RNA sequencing. Briefly, the first and second strand cDNA synthesis was performed using Strand Synthesis Reaction Buffer (5X) and DNA Polymerase I and RNase H, respectively. The blunt ends were purified with AMPure XP system (Beckman Coulter, Beverly, United States) after conversion of the exonuclease/polymerase activity and then used in PCR reactions, and library quality was assessed on the Agilent Bioanalyzer 2,100 system. Clean reads were obtained and used for all the downstream analyses. The DESeq2 R package (1.20.0) was used for searching DEGs in two conditions/groups by the standard of adjusted *p*-value ≤ 0.05. Corrected *p*-value of 0.05 and absolute foldchange of 2 were set as the threshold for significantly differential expression. Using the cluster Profiler R package to implemented the Gene Ontology (GO) enrichment analysis and Kyoto Encyclopedia of Genes and Genomes (KEGG) pathways of differentially expressed genes.

### Sample extraction and UPLC-ESI-MS/MS analysis of lipid and phenylpropanoid

For the metabolomic analysis, we chose the higher fertile P23 treated with 21°C and sterility P23 treated with 30°C, the obtaining method for differential fertility material for P23 is consistent with that described in 2.1. For lipid metabolism, we selected anthers of 11th stage for analysis using the UPLC-MS/MS method, with steps referring to our previous study (Sun et al., 2021).

For phenylpropanoid metabolism, freeze-dried (Scientz-100F) anther samples of 11th stage were crushed by mixer mill (MM 400, Retsch), then dissolve 100 mg of lyophilized powder with 1.2 ml 70% methanol solution, place the sample in a refrigerator at 4°C overnight. The sample extracts were analyzed using an UPLC-ESI-MS/MS system. The signal intensity of the characteristic ions was obtained in the detector. The integration and calibration of the chromatographic peaks, which represents the relative content of the corresponding substance, was performed using the MultiaQuant software, and finally all the metabolites are derived. Significantly regulated metabolites between groups were determined by VIP ≥ 1 and absolute log2FC (fold change) ≥ 1. Using R package MetaboAnalystR to generate the VIP values extracted from OPLS-DA result. The data was log transform (log2) and mean centering before OPLS-DA. To avoid overfitting, a permutation test (200 permutations) was performed.

### Qrt-PCR validate of transcriptome data and metabolism related gene expression

We selected the 9th, 10th, and 11th stages materials of P23 and 9th stage materials of P28 and P28F to extract total RNA. After checking the quality and concentration of RNA, the RNA samples were reverse transcribed into cDNA using the RevertAidTM First Strand cDNA Synthesis Kit. Specific primers were designed by Primer 5 and selected Acting7 as the internal reference gene were synthesized in Shenggong Bioengineering (Shanghai, China). The qPCR reaction system was 20 μl and quantitative analysis was performed using the QuantStudio™ Real-Time PCR Detection System with three biological replicates for each sample. Specific primer information is provided in Supplementary Table 3.

### Electron microscopy analysis of pollen, structure staining of pollen wall and determination of soluble sugar content

According to the experimental methods of previously studies, we selected 11 stage anthers for sample preparation and observation in scanning electron microscopy (SEM) and Transmission electron microscopy (TEM). These experiments were performed at the Electron Microscopy Center of Huazhong Agricultural University (Zhang et al., 2021). Mature pollen grains before flowering were selected, stained with 1% I_2_-KI for 5 min, and observed for starch accumulation under bright field in fluorescent microscope. The inner was stained with 0.1% Calcoflour white for 5–10 min and observed with a Fluorescein isothiocyanate (FITC) channel at 495 nm; the exine was stained with 0.001% Auramine O for 5–10 min and observed with an Ultraviolet (UV) channel at 180 nm. Soluble sugar content was performed using the plant soluble sugar content kit (KeMing, KT-2-Y, China).

### Statistical analysis and graphical plotting of data

The data in the study were statistical analyzed using SPSS for *t*-test, and images were drawn using Origin 2021. Cluster heat maps and VENN plots were plotted using TBtools, and KEGG and volcano plots were plotted using R. Graphical summary and pattern plots were stitched and drawn using Photoshop 2021 and Adobe Illustrator 2021.

## Results

### Recovery of pollen fertility under low temperature treatment

**Figure 1:**
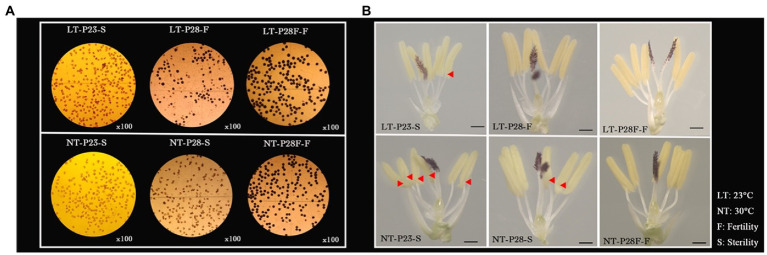
Pollen fertility **(A)** and anther phenotypes **(B)** of P23, P28, and P28F under different temperature treatments. LT/NT: 23°C/30°C treatment. F/S: Fertility/Sterility anthers. The red triangle indicates the position of the malformation in the sterile anther.

First, the NILs of the rice PTGMS line (P23 and P28) and conventional rice (P28F) with the same genetic background were treated at different temperatures to obtain plants with differential fertility among the three lines. As shown in Figure 1A, P23 remained sterile under the low temperature 23°C treatment, while P28 was partially restored to fertility and P28F was fully fertile. However, the pollen grains from P23 and P28 were completely sterile under the high temperature 30°C treatment, except for P28F which showed complete fertility. We found that the 23°C treatment restored the fertility of P28 pollen to 49.61% and its seed setting rate to 41.55%. The iodine staining rate and seed setting rate of P28F under different temperature treatments were 85.24%–91.80% (Figure 1A; Table 1). It was also observed that fertile pollen grains were deeply stained in P28 and P28F and with no staining in P23 pollen grains. The sterile anthers of P23 were clearly deformed (red triangles), with small anthers and white color, while the anthers of P28 and P28F fertile plants were rounded and had a yellow color. The anthers of P28 sterile plants were not significantly deformed, and the color was white (Figure 1B). However, it was observed that the anthers treated with LT were smaller than those treated with NT, and there was no significant change in anthers between the three NILs in the same treatment. This result indicates that low-temperature treatment below the critical temperature restored male fertility in rice PTGMS lines, the critical temperature for fertility in the three experimental lines was stable, and the material for fertility differences was reliable.

**Table 1:** Pollen fertility and seed-setting rate of P23, P28, and P28F.

Materials	Treatments	Pollen I_2_-KI staining rate	Seed setting rate
		Mean ± SD (%)	Mean ± SD (%)
P23	LT (23°C)	0.00	0.00
	NT (30°C)	0.00	0.00
P28	LT (23°C)	39.61 ± 0.25^*^	41.55 ± 1.25^*^
	NT (30°C)	0.00	0.00
P28F	LT (23°C)	91.80 ± 0.88	85.24 ± 1.01
	NT (30°C)	90.08 ± 0.76	88.79 ± 1.70

Value is expressed as mean ± standard deviation, *n* > 120; asterisks indicate significant differences revealed by Student’s *t*-test at *p* < 0.05 (*).

### Comparative transcriptome analysis and differentially expressed gene identification analysis under different temperature treatments

Transcriptome analysis was performed on P23, P28, and P28F treated with LT and NT, and a total of 18 libraries were prepared for analysis. A summary of the sequencing and mapping results is shown in Supplementary Table 1. A resulting showed that 790 million clean reads were obtained with an average Q30 value of 93.08%. Then the clean reads were mapped to the reference genome, and there were 91.88–94.12 reads were mapped to the genome. To assess the consistency between biological replicates of each sample, a clustering heatmap was drawn based on the correlation coefficient between each sample (Supplementary Figure 2). The results indicated that the replication between the three biological replicates of each sequencing sample was excellent with *R*^2^ > 0.96. However, the bipolar classification of LT/NT or F/S did not appear in the clustering analysis, which indicated that the gene expression resulted from the combined effect of temperature changes and fertility regulation.

**Figure 2:**
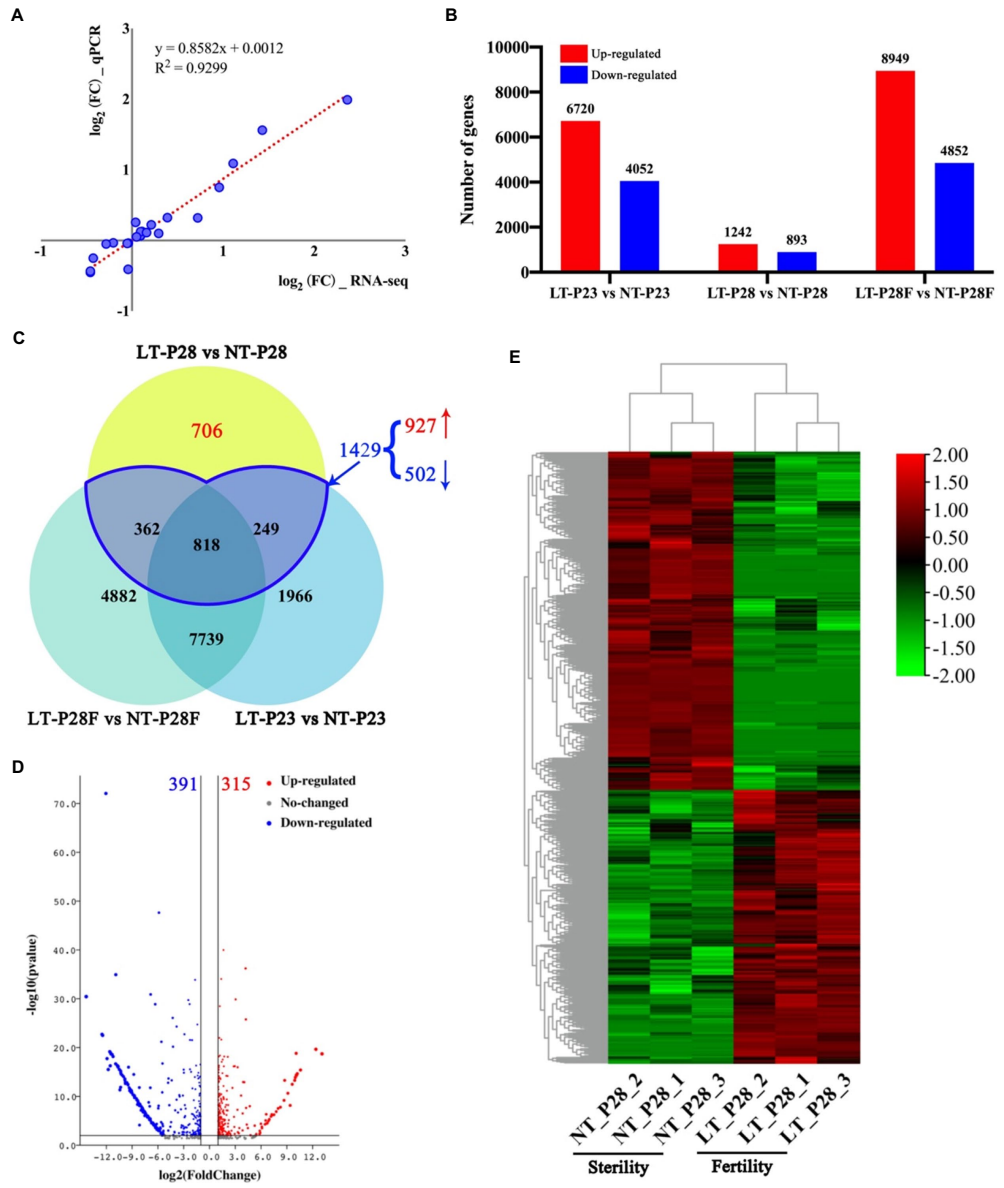
Analysis of DEGs in P23, P28, and P28F under different temperature treatments. **(A)** Quantitative real-time PCR (qRT-PCR) assay of sequencing data. **(B)** Number and up-regulation and down-regulation relationships of DEGs in P23, P28, and P28F under different temperature treatments. **(C)** VENN plots of DEGs in P23, P28, and P28F under different temperature treatments. **(D)** Up-regulation and down-regulation relationships of DEGs in response to changes in fertility. **(E)** Expression heat map for clustering of DEGs in response to changes in fertility. LT/NT: 23°C/30°C treatment. F/S: Fertility/Sterility plants. The red bars indicate up-regulated expressed genes and the blue bars indicate down-regulated expressed genes in **B**.

Transcriptome sequencing revealed significant differences in gene expression between samples by temperature treatment. First, eight genes were randomly selected among the six samples for qRT–PCR analysis. Correlation analysis showed that the RNA-seq results were highly correlated with the qRT–PCR results, with a Pearson coefficient of 0.93 (Figure 2A). The results indicated that the results of RNA-seq were accurate. To identify DEGs that respond to temperature changes and involved in regulating male fertility, we performed DEG analysis on samples obtained under the LT and NT treatments, respectively. First, we performed a comparative analysis of gene expression in the three lines under different temperature treatments. A total of 10,772 differentially expressed genes were found in LT-P23 compared with NT-P23, of which 6,720 were upregulated and 4,052 were downregulated. Similarly, there were 2,135 and 13,801 differentially expressed genes in the comparison groups of P28 and P28F, respectively. There were 1,242 and 8,949 upregulated genes and 893 and 4,852 downregulated genes, respectively (Figure 2B). Differentially expressed genes were responsive to temperature changes in three NLs treated with 23°C and 30°C. However, only P28 showed differential changes in fertility, which indicated the presence of differentially expressed genes in P28 that responded to temperature changes and were involved in male fertility regulation. VENN plot analysis revealed that the DEGs shared with P23 and P28F in P28 are specifically responsive to temperature changes (362, 818, and 249). Among the 1,429 DEGs, 927 were upregulated and 502 were downregulated in P28. We obtained 706 genes involved in male fertility regulation in P28 after removing DEGs that only responded to temperature changes. Of these, 315 genes were upregulated and 391 genes were downregulated in expression (Figure 2D). The clustered heatmap showed a high degree of consistency in the expression of different genes in the differential fertility material, and the results of clustering also matched with the male fertility traits (Figure 2E). These results indicate that the DEGs involved in the regulation of male fertility in P28 obtained by comparative analysis are accurate.

### Go and KEGG enrichment analysis of degs in response to male fertility

**Figure 3:**
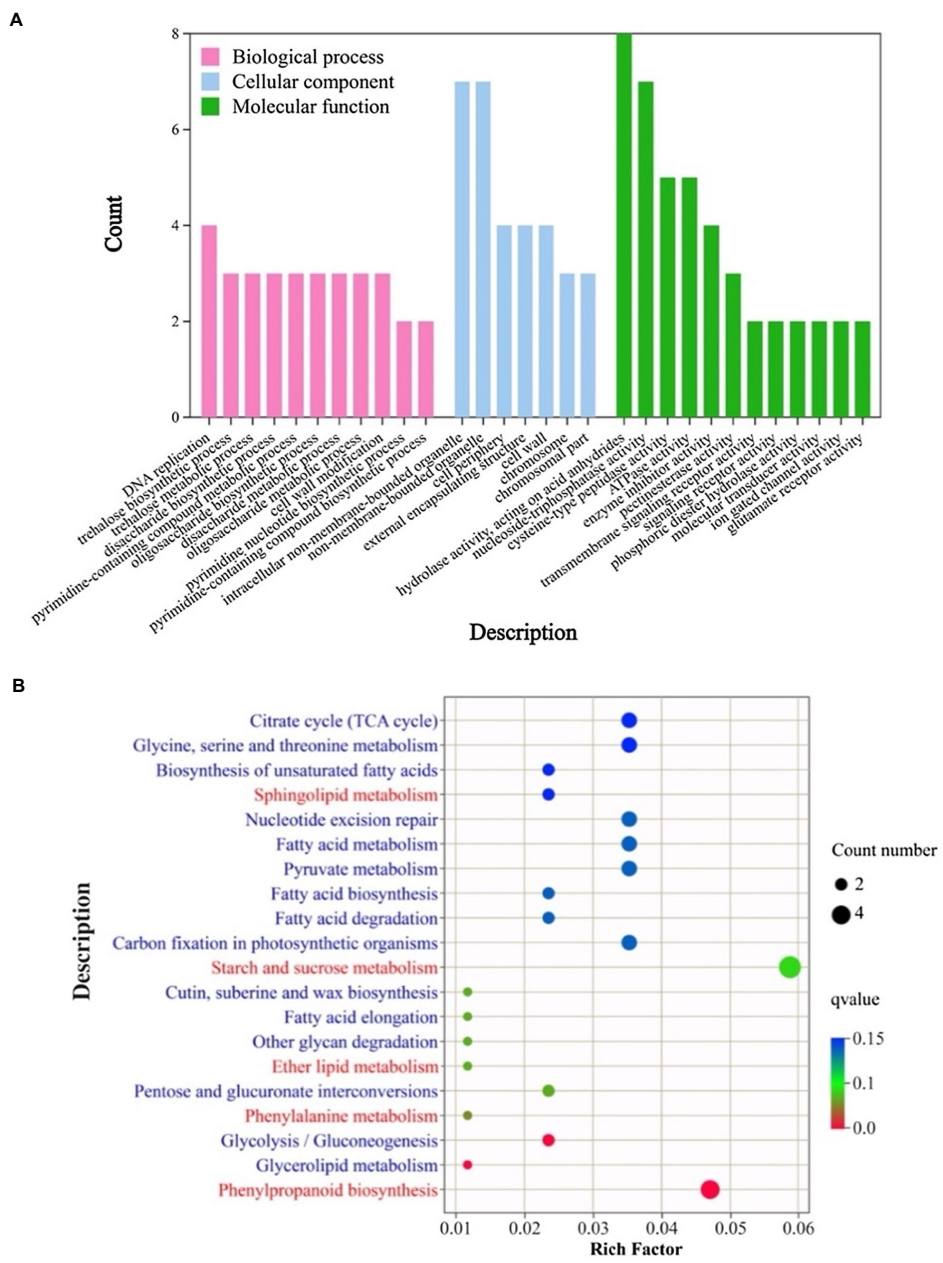
GO enrichment analysis **(A)** and KEGG enrichment analysis **(B)** of the DEGs in response to male fertility. In **A**, the *x*-axis represents different subcategories, while the *y*-axis indicates the count of genes of the corresponding GO terms. BP, biological processes; CC, Cellular component; MF, molecular functions. In **B**, the *x*-axis represents the enrichment factor, while the *y*-axis indicates the description of the KEGG pathway.

We are interested in whether the same differences in metabolic pathways are present in DEGs that respond to temperature changes and are involved in fertility regulation. The 706 DEGs involved in male fertility were assigned to 46 GO classes/terms with GO annotation analysis. We present the top 30 biological processes (BP), cellular component (CC) and molecular functions (MF) in Figure 3A. Under the classification of biological processes, “trehalose biosynthetic process,” “trehalose metabolic process,” “disaccharide biosynthetic process,” “disaccharide metabolic process,” “oligosaccharide biosynthetic process,” “oligosaccharide metabolic process” and “cell wall modification” were prominently represented. The GO terms enriched in the cellular component classification were “nonmembrane-bounded organelle,” “cell wall” and “cell periphery.” “Transmembrane signaling receptor activity,” “phosphoric diester hydrolase activity” and “pectinesterase activity” were the most enriched GO terms in the molecular function. The results of GO enrichment analysis indicated that the processes of carbohydrate metabolism, lipid metabolism and cell wall composition are closely associated with male fertility regulation.

The biological pathways involved in the process of male fertility change were identified using KEGG pathway enrichment analysis, and the biological pathways of the top 20 are shown in Figure 3B. These metabolic pathways were mainly enriched in lipid metabolism processes, such as “sphingolipid metabolism,” “ether lipid metabolism” and “fatty acid metabolism.” Meanwhile, the metabolic pathways of sugar were mainly enriched with metabolic processes of “starch and sucrose metabolism” and “pentose and glucuronate interconversions.” In addition, the “phenylpropanoid biosynthesis” and “phenylalanine metabolism” metabolic pathways were significantly enriched. The results of the KEGG enrichment analysis indicated that lipid metabolism, sugar metabolism and phenylpropanoid metabolic processes were involved in the process of male fertility change in rice, which together determined male fertility.

Meanwhile, we performed KEGG analysis on DEGs that only responded to temperature changes (1,429). Here, there were more metabolic pathways significantly enriched, such as “phenylpropanoid biosynthesis,” “galactose, Starch and sucrose metabolism,” “flavonoid biosynthesis” and “sphingolipid metabolism” (Supplementary Table 2). These results indicated that temperature changes altered gene expression in sugar, lipid and phenylpropane metabolic pathways, some of which produce qualitative changes in DEDs (706) involved in male fertility regulation.

### Abundant sugar metabolic processes and complete pollen wall structure in fertile anther

After obtaining the metabolic pathways enriched with differentially expressed genes, we were interested in the differences in sugar metabolism and pollen wall synthesis in pollen. Based on the results of our experiments over many years, we find that the fertility results of P23 anthers are more stable and more fertile than those of P28 anthers under low-temperature treatment (Supplementary Table 4). Therefore, we obtained fertility and sterility anther of P23 for cytological observation and metabolomic analysis by treatment at 21°C and 30°C, respectively (Supplementary Figure 3).

**Figure 4:**
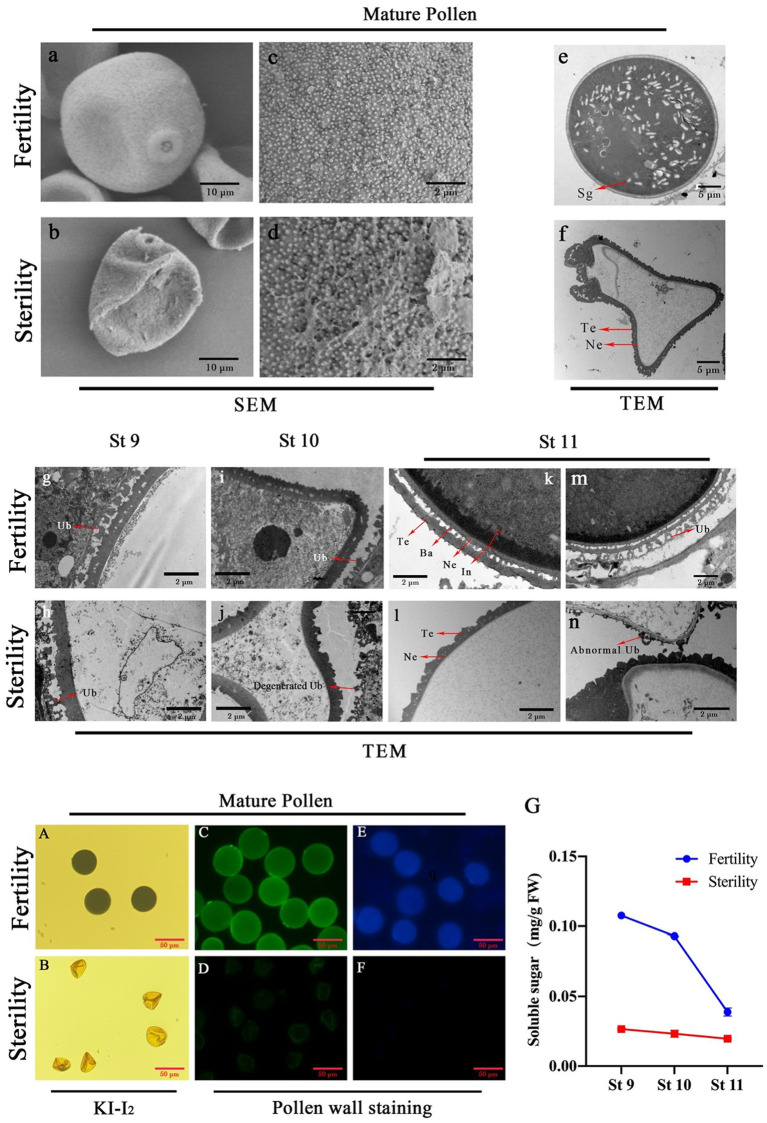
Observation of pollen grain structure *via* electron microscopy and staining. Electron microscopic observation of fertility **a, c, e** and sterility **b, d, f** pollen grains. TEM observation of the pollen wall structure in fertility **g, i, k, m** and sterility **h, j, l, n**. Pollen stained by KI-I_2_ **A, B**, Auramine O **C, D** and Calcofluor White **E, F**; Soluble sugar contents **G** in fertility and sterility plants. Ub, Ubisch body; Te, texine; Ba, bacula; Ne, nexine; In, intine; Sg, starch granule.

First, SEM of mature pollen grains showed that the fertile pollen grains were fuller and the sporopollenin was evenly distributed on the surface of the pollen grains compared to the sterile pollen grains, while the sterile pollen grains were hollow and had a rough surface structure (Figures 4a–d). TEM observation of mature pollen grains revealed that there are a large number of starch granules (Sg) filling in fertile pollen grains, while sterile pollen grains did not have any substantial filling (Figures 4e,f). Compared to sterile pollen grains, fertile pollen grains were round and fully filled, while sterile pollen grains were hollow and deflated. Subsequently, we observed the fertility differences in anthers at stages 9, 10, and 11 using TEM and found that the pollen wall structures of fertile and sterile anthers are similar at stages 9 and 10. At stage 11, there were clear three-layer exine and inner structures in fertile anthers and the starch grains began to accumulate. However, there was no normal pollen wall structure and substance accumulation in the sterile anthers. Texine (Te) and nexine (Ne) were muddled in the exine of sterile pollen grains, and there was an absence of bacula (Ba) structure (Figures 4g–l). The ubisch body in sterile anthers was significantly degraded at stage 11 compared to that in fertile anthers (Figures 4m–n). This result indicates that an abnormal starch accumulation and pollen wall development in sterile pollen grains, and that the 11 stage is a critical stage for pollen wall development and fertility formation. Subsequently, the results of starch iodine staining showed a high accumulation of starch in fertile pollen grains, and strong fluorescence staining on the inner and exine regions of pollen; meanwhile, no iodine staining was found in sterile pollen grains, and there was a weak fluorescence signal on the exine, while the inner regions did not exhibit any fluorescence (Figures 4A–F). The soluble sugar content was higher in fertile anthers than in sterile anthers and showed a sudden drop at stage 11 (Figure 4G). These results demonstrated that the lack of inner wall structures and normally developing exine in sterile pollen grains, and the 11 stage is a critical stage for metabolite transformation and accumulation in pollen grains.

### Increased accumulation of lipid metabolites in fertile plants

We found that the 11 stage is the key stage of pollen wall formation and content filling in pollen grains base on the results of electron microscopic observations and soluble sugar changes, it indicating that anthers undergo accumulation and conversion of metabolites at stage 11. Therefore, we evaluated the metabolite level differences in anthers.

**Figure 5:**
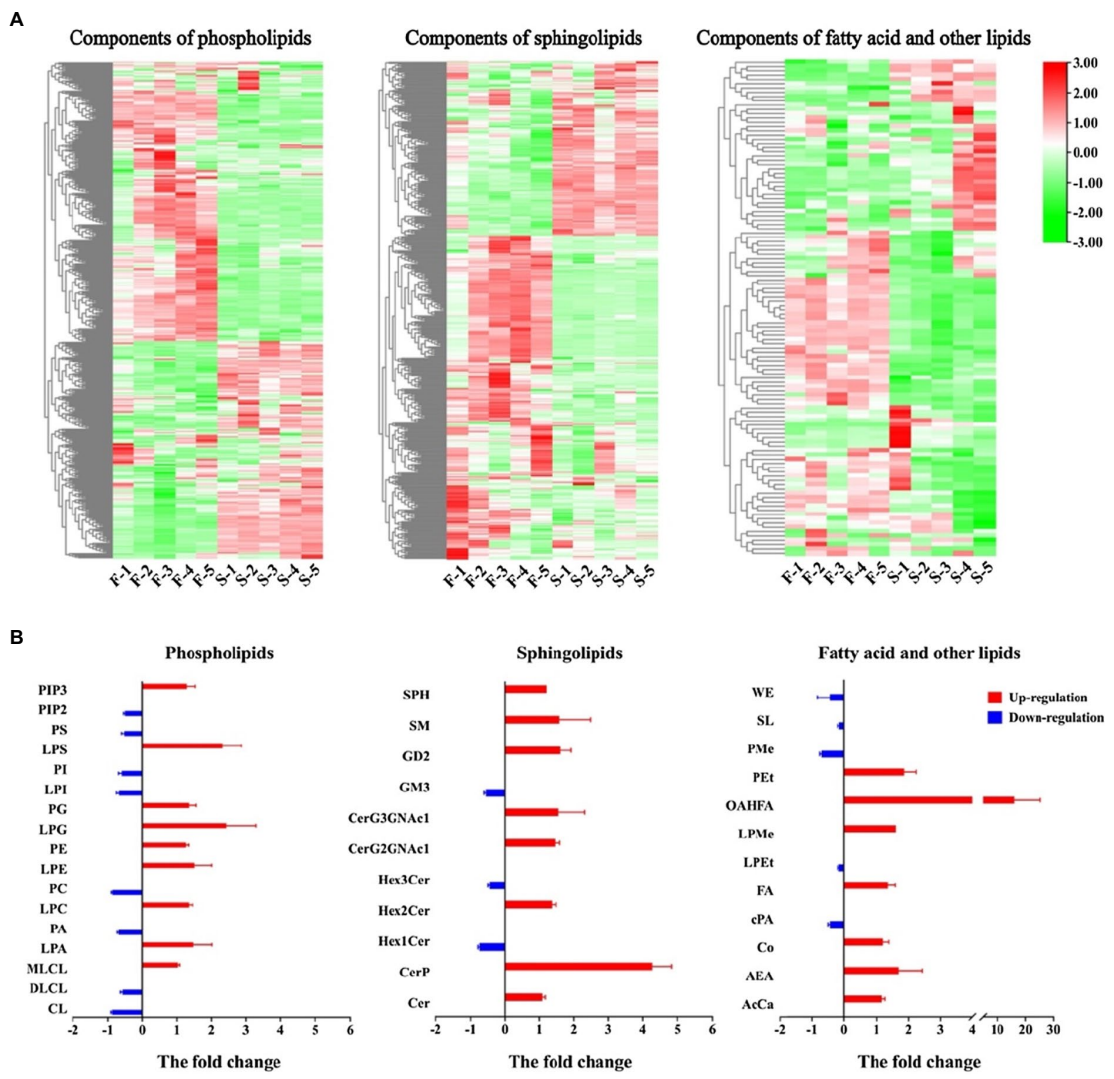
Differences in lipid metabolites were compared in fertile and sterile anthers. **(A)** Cluster heat map of different components in lipids. **(B)** The change of phospholipids, sphingolipids, fatty acids and other lipids in the comparative group. In **B**, the *Y*-axis indicates the different lipids and the *X*-axis indicates the fold change of lipid in the comparison group of fertile and sterile plants. Red bars indicate that the lipid is upregulated in fertile plants and blue bars indicate that the lipid is downregulated in fertile plants. The full names of the abbreviations in the classification were shown in the abbreviated list (Supplementary Table 5).

Fatty acids and their derivatives are essential components of anther sporopollenin, cuticle and pollen wall formation. Some lipid metabolites were classified by categorization analysis into three major groups: phospholipids, sphingolipids and fatty acid and other lipids. The variation in the content of each lipid component in the classification of differentially fertile plants is shown in Figure 5A. Many components of the three categories were upregulated in fertile plants. More than 65% of the sphingolipids and fatty acids and other lipids were significantly upregulated. The statistics of the components were found that 17, 11, and 12 secondary categories were present in the three classifications, respectively (Figure 5B). Nine of the phospholipid components in fertile plants were upregulated and eight were downregulated. Among these, the fold change of LPS and LPG were >2. Eight of the sphingolipids were upregulated in fertile plants and Cerp differences reached fourfold. Similarly, seven of the fatty acids were upregulated in fertile plants, and the OAHFA differed up to 20-fold. These results of the differences in lipid metabolism indicated a more active accumulation of lipid in fertile plants and its essential for the formation of pollen wall and male fertility.

### Differences in metabolites of flavonoids and lignin affect male fertility formation

**Figure 6:**
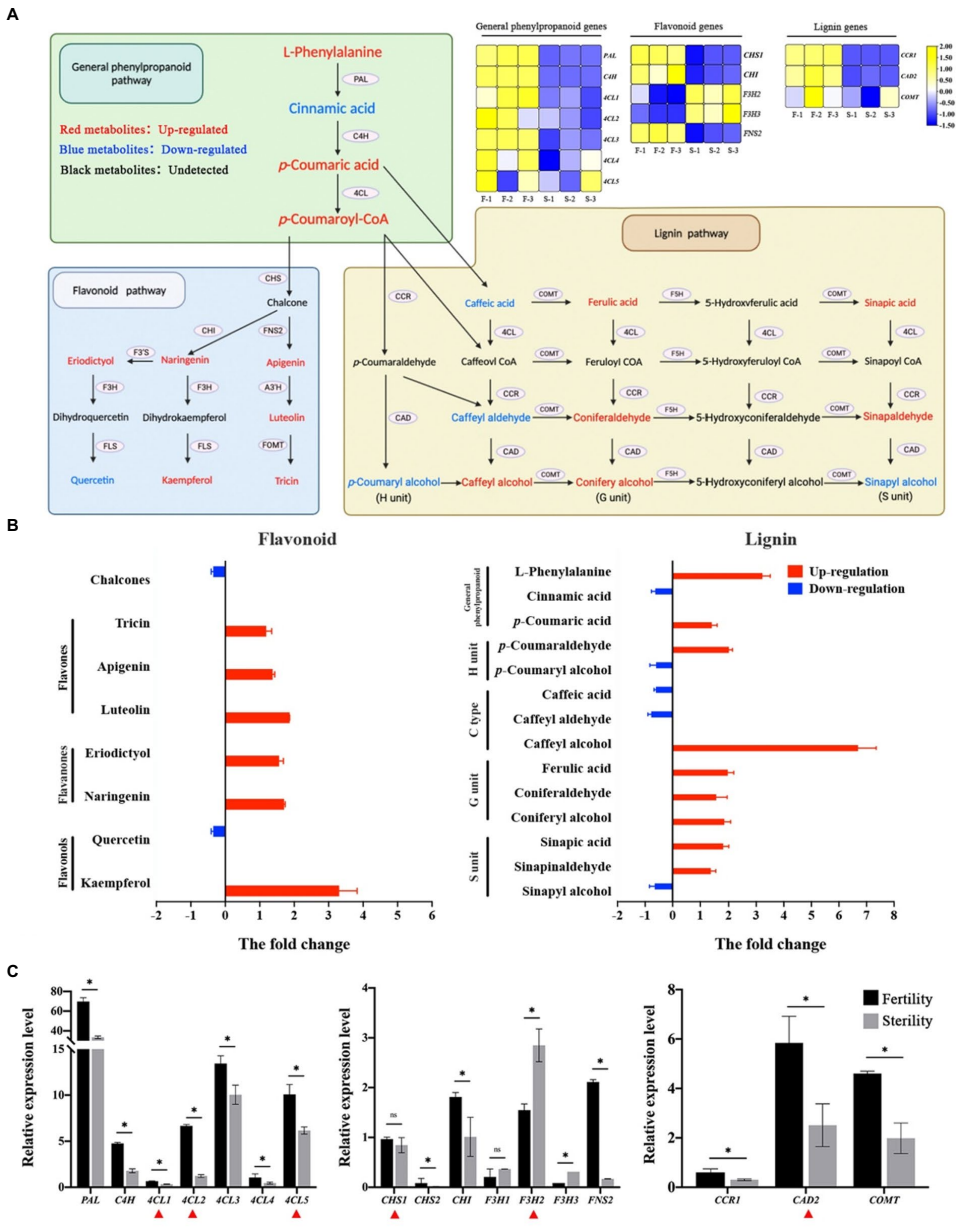
Differences in phenylpropanoid metabolism were compared in fertile and sterile anthers. **(A)** Metabolic pathways and critical gene expression of phenylpropanoid in rice. **(B)** The changes of flavonoids and lignin metabolites in the comparison group. **(C)** Expression of critical genes in phenylpropanoid metabolism in fertile and sterile anthers by qPCR. In **A**, the Heatmap shows the transcriptome data of critical genes. Red metabolites indicated up-regulated substances and blue metabolites indicated down-regulated substances. In **B**, the *Y*-axis indicates the different substances and the *X*-axis indicates the fold change in the comparison group of fertile and sterile plants. Red bars indicate that the substance is upregulated in fertile plants and blue bars indicate that the substance is downregulated in fertile plants. In **C**, the qPCR results show the average of three replicates. ^*^p < 0.05. ns, Not significant. The red triangles indicate the DEGs in P28.

The pollen exine is composed of sporopollenin, and phenylpropanoid pathway derivatives are essential for synthesizing sporopollenin and pollen wall development in plants (Wang et al., 2020; Xue et al., 2020). The abnormal sporopollenin distribution and pollen exine structure suggested that we perform a phenylpropanoid metabolome analysis. It is divided into three parts according to the metabolic pathway: general phenylpropanoid pathway, flavonoid pathway and lignin pathway (Figure 6A). According to the transcriptome data, the phenylalanine ammonia lyase (*PAL*), cinnamate 4-hydroxylase (*C4H*) and 4-coumarate:coenzyme A ligase (*4CL*) genes involved in the general phenylpropanoid pathway were upregulated in fertile plants. However, the flavanone 3-hydroxylase (*F3H*) gene expression trend in the flavonoid pathway was downregulated as opposed to chalcone synthase (*CHS*), chalcone isomerase (*CHI*) and flavone synthase II (*FNS2*). Flavonoid metabolomics results revealed that three components (flavones, flavanones, and flavonols) were significantly increased in fertile anthers (Figure 6B). The fold change in flavonols was >3 for kaempfero, and quercetin was downregulated in fertile anthers. In contrast, the other two types of flavonoids (flavones and flavanones) were upregulated in fertile anthers. L-phenylalanine was significantly up-regulated in fertile anthers according to the lignin differential metabolite analysis. Among the three lignin constituent units, coniferyl alcohol was upregulated in fertile anthers, which constitute the G units; however, caffeyl alcohol is a C-type lignin substance with a difference fold of seven. The results of metabolome analysis indicated that flavones and flavanones are the major contributors to the exuberant flavonoid metabolism in fertile anthers; the G units and C-type lignin are the major lignin units that contribute to the formation of male fertility. Finally, gene expression validation in fertile and sterile anthers revealed that all genes of the phenylpropanoid metabolic pathway were upregulated in fertile anthers, except *F3H* (Figure 6C); among them, the expression of *4CL1, 4CL2, 4CL5, CHS1, F3H2* and cinnamyl alcohol dehydrogenase 2 (*CAD2*) was significantly different in P28 (red triangle in Figure 6C). These results suggested that active flavonoid metabolism and lignin synthesis are characterized among the levels of gene expression and secondary metabolites, and which make positive contributions to the regulation of male fertility.

### Differentially expressed genes in the sugar, lipid, and phenylpropanoid metabolic pathways might be essential for the formation of male fertility

**Figure 7:**
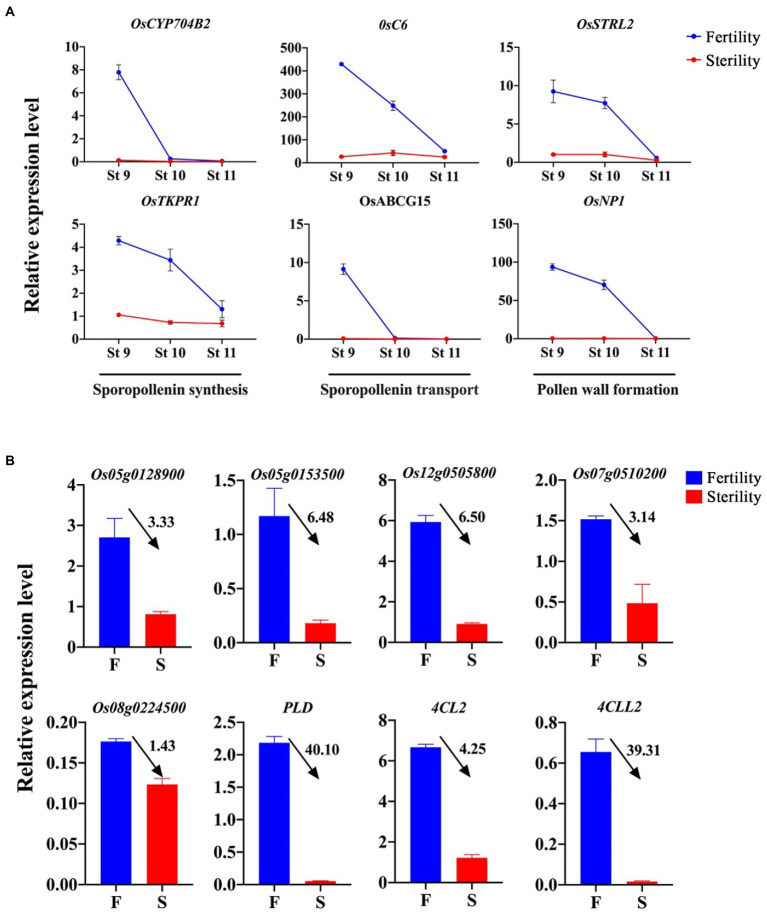
Expression of genes involved in pollen fertility formation and potential male fertility regulation. **(A)** Expression of genes for sporopollenin synthesis, sporopollenin transport and pollen wall formation. **(B)** Expression of inferred related genes involved in the regulation of male fertility. In **B**, numbers indicate the fold change of expression in fertility differential anthers.

To further understand the differences in sporopollenin and pollen walls in differentially fertile anthers, the expression of several known functional genes in rice was compared using RT–qPCR (Figure 7A). These genes are involved in the processes of sugar, lipid and phenanthrene metabolism (Li et al., 2010; Zhang et al., 2010; Zhao et al., 2015; Liu et al., 2017; Zou et al., 2017; Xu et al., 2019) The results showed that *OsCYP704B2*, *OsTKPR1*, *OsC6*, and *OsABCG15*, which are the essential genes in the synthesis and transport of sporopollenin precursors, were significantly upregulated in fertile anthers. The same trends appeared in *OsSTRl2* and *OsNP1*, the critical genes in pollen wall formation. These results demonstrate that normal sporopollenin synthesis and transport processes in fertile anthers ensure the building of pollen walls.

Finally, we performed expression analysis of genes enriched in the pathway for sugar, lipid and phenylpropanoid metabolic processes involved in the regulation of male fertility (Figure 7B), and these genes respond to temperature changes and are involved in regulating male fertility. *Os05g0128900*, *Os05g0153500*, and *Os12g0505800* are proteins encoding trehalose 6-phosphate phosphatase, and *Os07g0510200* and *Os08g0224500* are proteins encoding beta-1,3-glucanase. These genes are involved in trehalose biosynthesis and beta-1,3-glucan hydrolysis processes within carbohydrate metabolism. *PLD* is the protein encoding phospholipase D, which acts in the process of lipid catabolism. In addition, *4CL* and *4CLL2* are proteins encoding 4-coumarate-CoA ligase (4CL), serving in the synthetic pathway of phenylpropanoid metabolites. Gene expression analysis revealed that these genes were upregulated in fertile anthers compared to sterile anthers, with a sixfold difference in *Os05g0153500* and *Os12g0505800*. The same expression trend was observed in *PLD*, *4CL*, and *4CLL2*, with a nearly 40-fold difference in *PLD* and *4CLL2*. This result suggests that sugar metabolism, lipid metabolism and phenylpropanoid metabolism are essential processes involved in the formation of male fertility in rice, in which some key genes are potential regulators of fertility regulation.

## Discussion

Rice PTGMS lines serve as seed production materials for two-line hybrid rice, in which male fertility is controlled by temperature and light conditions. However, the variability and instability of the environment present a huge challenge to two-line hybrid seed production. Therefore, it is necessary to understand the male transition mechanism and explore the regulatory factors in rice PTGMS lines. In this research, we compared phenotypic, genomic and metabolite differences in plants with differential fertility by performing phenotypic analysis, comparative transcriptome analysis and metabolome analysis among the NILs and exploring the metabolites and potential regulatory genes involved in the male fertility regulation process.

### Abnormal pollen wall development and male sterility in rice PTGMS lines induced by high temperature

Photoperiod- and thermosensitive genic male sterility lines rice are sensitive to temperature. Male sterility exhibits at temperatures above the critical temperature and regained male fertility is regained at temperatures below the critical temperature (Li et al., 2020). This study found that the two rice PTGMS lines exhibited male sterility and lack of starch accumulation in pollen grains when subjected to a temperature treatment <30°C, with slight differences in anther phenotype (Figure 1). The reproductive stage of rice is usually more sensitive to temperature than the nutritional stage. High temperatures can affect the processes of anther dehiscence and pollen grain swelling during the gametogenesis, reducing seed setting rates and affecting crop yield (Matsui and Omasa, 2002; Wahid et al., 2007; Chen et al., 2020). The most important event after microspore meiosis is the synthesis of the pollen wall and the pollen shell. Sporopollenin is a highly resistant biopolymer composed of lipids released by the tapetum and constitutes the exine of the pollen. It has been found that genes such as *DPW2*, *OsTKPR1*, *CYP703A3*, *CYP704B2*, and *OsABCG26* regulate the production or transport of sporopollenin, which contributes to pollen wall development. These genes are involved in pollen wall formation by regulating the synthesis or transport of sporopollenin (Li et al., 2010; Yang et al., 2014; Zhao et al., 2015; Xu et al., 2016), and their mutants exhibit abnormal pollen wall structure and a male sterile phenotype. The sterile plants in this study also showed abnormal exine of pollen grains at high temperatures, while the absence of inner structure led to the nonaccumulation of starch and sterility of pollen. Compared with the inner structure, the exine of sterile pollen grains lacks a Ba structure and the outer side of the exine lacks a Ub structure. Ub is involved in sporopollenin synthesis, and the presence of Ub is directly related to pollen wall formation in the *TIP3* mutant (Yang et al., 2019). Therefore, the absent Ub may be a direct phenotype of the abnormal process of sporopollenin precursor substance synthesis. Since the formation of Ub precedes that of pollen wall, the coexistence of normally developing Ub and sterile pollen grains in some mutants is observed (Zhang et al., 2020). In addition, sporopollenin synthesis transport and pollen wall formation genes were significantly downregulated in male sterile anthers induced by high temperature (Figure 7A). Therefore, we suggest that abnormal pollen wall development induced by high temperature is the primary reason for male sterility in rice PTGMS lines.

### The active metabolic processes of sugar, lipid, and phenylpropanoid are essential for the formation of male fertility

In addition to pollen wall defects, the most intuitive phenomenon is the absence of the accumulation of any starch observed in the iodine staining and TEM of the sterile pollen grains (Figure 4). The starch content in pollen grains can be used as an indicator of pollen viability and maturity (Song et al., 2001). Abnormal pollen development and low starch accumulation were found in the sugar metabolism defective mutants *osuam3*, *oscap1*, and *osgt1*. These genes encode UDP-arabinopyranose mutase 3, arabinokinase-like protein and glycosyltransferase, respectively, which are important components of polysaccharide biosynthesis and metabolic processes (Moon et al., 2012; Ueda et al., 2013; Sumiyoshi et al., 2014). Therefore, defects in sugar metabolism during pollen development can result in male sterility. Sugar metabolism includes the biosynthesis, degradation and transport of sugars, which are transported into the anther during the reproductive stage to provide the substance basis for callose wall and primexine development, inner wall formation, starch accumulation and pollen maturation in the microspore (Shi et al., 2015). In this study, a significant accumulation of starch was observed in fertile pollen grains, while the content of soluble sugars was twice as high as that of sterile pollen walls. Therefore, higher levels of sugar metabolism are essential for the formation of male fertility.

The anther cuticle and pollen wall are composed of fatty acids and their derivatives, and any disruption in these two structures usually results in microspore abortion and male sterility (Zhang et al., 2016). Some studies have found that lipid metabolism primarily occurs in the tapetum of anthers, but increasing evidence confirms that this process is also present in the anther epidermis (Zhan et al., 2018; Wan et al., 2019). The pollen wall accumulates sporopollenin, and its development usually shares some lipids with epidermal growth (Jiang et al., 2013). Genes related to lipid metabolism, such as fatty acyl-CoA synthetase (*ACOS*), cytochrome P450 fatty acid hydroxylase (*CYP703A*), long-chain fatty acid ω-hydroxylase (*CYP704B*) and GDSL lipase have conserved functions in controlling plant development of the pollen wall and anthers (Wan et al., 2020). Phospholipid signalings is involved in pollen development, and storage oil bodies with triacylglycerol (TAG) as the main component can provide energy for pollen grain maturation (Piffanelli et al., 1998). Sphingolipids can act as second messengers and participate in the control of programmed cell death in the tapetum (Bleicher and Cabot, 2002). In this study, the major component of phospholipids, PE, was significantly upregulated in fertile anthers and the basic structures of sphingolipids, Cer, and CerP. In addition, seven fatty acids contributed positively to fertile formation (Figure 5). This result also indicates that active lipid accumulation provides the substance basis for constructing sporopollenin and pollen walls.

The phenylpropanoid pathway provides intermediates for synthesizing lignins and flavonoids, which are major metabolic processes in plant growth and development. Previous studies have demonstrated that the phenylpropane metabolic pathway is regulated by temperature changes, especially the flavonoid metabolic process (Solecka et al., 1999; Guo et al., 2020; Hao et al., 2021b; He et al., 2021c). Flavonoids have also been demonstrated to have a functional role in plant male fertility (Wang et al., 2020; He et al., 2021b), and it was found that impaired pollen function and reduced seed setting appeared in a plant bearing the *chs* mutant and the RNAi-mediated suppression of *FLS* gene (Burbulis et al., 1996; Mahajan et al., 2011). In Nipponbare, it was found that *OsCHI* mutation led to a decrease in seed yield, and the mutation of *CHI* usually led to a large depletion of various flavonoid accumulation in plants, which indicated a positive correlation between *CHI* and flavonoid content (Hirano et al., 2017; Clayton et al., 2018). In this study, *CHS*, *CHI*, and *FNS2* were upregulated in fertile anthers, whereas all three flavonoids were upregulated in fertile anthers except quercetin. Therefore, the main contributing flavonoids to fertile anther formation may be flavones and flavanones. In addition, FNS2 and A3′H/C5′H produce tricin during lignification in rice, and tricin can function as comonolignol to form integrated lignin (Lan et al., 2015). Similarities between lignin in pollen walls and xylem cell walls have been demonstrated, and the synthesis of sporopollenin in pollen requires the involvement of lignin metabolism in the phenylpropanoid pathway, and researchers have detected some phenolic components (*p*-coumaric acid and ferulic acid) in sporopollenin (Li et al., 2019a; Xue et al., 2020). In *Arabidopsis*, mutations in several key genes in the lignin synthesis pathway result in defective lignin synthesis and male sterility, for example, the *c4h* and *ccr1* single mutants, the *4 cl1 4 cl2 4 cl3* triple mutant, and the *pal1 pal2 pal3 pal4* quadruple mutant (Mir Derikvand et al., 2008; Schilmiller et al., 2009; Huang et al., 2010; Li et al., 2015). In this study, we found that the G unit was significantly upregulated in fertile anthers among the three lignin units, while Xue et al. (2020) found the presence of the lignin G unit, *p*-BA, *p-*coumaric acid, and ferulic acid in sporopollenin, and the S unit was not detected. This result was similar to ours, and we found that C-type lignans were also significantly up-regulated in fertile anthers. In addition, up-regulated expression of *CAD2* in fertile plants acts in the last step of lignin monomer synthesis, while significant up-regulation of G units and C-type lignin indicated that they might be specifically regulated by *CAD* in pollen wall development. We suggested that lignin G unit and C-type lignin are the primary substances involved in pollen wall formation, and the relatively active synthesis process provides the basis for the formation of pollen walls and fertility.

### Genes related to the sugar, lipid, and phenylpropanoid metabolic pathways are involved in the regulation of male fertility in rice

Cellular degradation of the tapetum exports essential nutrients (sugars and lipids) to the microspores for their early development. The callose enzyme (β-1,3-glucanase) signals for pollen wall development, and its degradation of callose promotes the release of microspores from the tetrads. Some studies have revealed that silencing the gene encoding β-1,3-glucanase in rice disrupts the callose degradation process and produces male sterility (Wan et al., 2011). In this study, comparative transcriptome analysis revealed that *Os07g0510200* and *Os08g0224500*, the genes encoding beta-1,3-glucanase, were significantly enriched in the sugar metabolism pathway; they were significantly upregulated in fertile anthers, with 3.14- and 1.43-fold higher expression than in sterile anthers, respectively. Higher levels of β-1,3-glucanase in fertile anthers promote the timely degradation of callose, which is a prerequisite for forming male fertility in rice.

Genetic analysis in plants has shown that trehalose is essential for carbohydrate metabolism and plant development. Trehalose is involved in the regulation of nutritional growth and organ development as a signalling molecule synthesized from UDP-glucose and glucose 6-phosphate under the action of trehalose-6-phosphate synthase (TPS) and trehalose-6-phosphate phosphatase (TPP), and TPS acts on TPP to remove the phosphate from it to produce free trehalose (Junior et al., 2013; Lastdrager et al., 2014). Low-temperature treatment increased the expression of TPP and the level of alglucan in rice, and its synthesis process was regulated by temperature changes (Habibur Rahman Pramanik and Imai, 2005). Some studies have found that in the genic male sterile line land cotton, TPP and TPS proteins in trehalose biosynthesis are significantly downregulated in sterile anthers; in addition, nine trehalose synthesis-related genes were differentially expressed in differentially fertile anthers of wheat. Therefore, it is believed that abnormal synthesis of trehalose causes pollen abortion (Yue et al., 2014; Li et al., 2019b; Hao et al., 2021a). In *Arabidopsis*, loss of function of *AtTPS1* also leads to altered cell wall structure and embryonic lethality (Gómez et al., 2006). In this study, three TPP encoding genes, *Os05g0128900*, *Os05g0153500*, and *Os12g0505800*, were enriched in the sugar metabolism pathway and showed a significant difference in their 3–6 fold in anthers with differentially fertility. In conclusion, we suggest that TPP genes are induced by temperature and have the potential to regulate male fertility and that they may be an important contributor to the process of starch accumulation and pollen grain formation in fertile anthers.

Disruption of lipid metabolism during anther and pollen development usually results in male sterility, and at least 82 genes related to lipid metabolism have been identified that determine male fertility and involved in the formation of the anther cuticle and pollen wall (Wan et al., 2020). Phosphatidic acid (PA) is an intermediate in lipid metabolism that can stabilize actin in the cytoplasm and is involved in pollen tube vesicle transport, pectin deposition and cell wall formation (Chen et al., 2018). Phospholipase D (PLD) is a functional enzyme in which the expression level and enzymatic activity are activated during low-temperature acclimation (Wang et al., 2006; Wan et al., 2009). It regulates PA production and degradation by cleaving phospholipid structures and releasing PA and free polar headgroups. It has been suggested that PLD activity is essential for the regulation of pollen tube growth. The decrease in PA levels disrupts membrane transport and alters the deposition of pectin and callose on the cell wall (Potocký et al., 2003; Pleskot et al., 2012). Some studies have found that PLD is highly expressed in late anther development, and might be involved in male sterility by regulating anther dehiscence (Ma et al., 2021). However, downregulation of *PIP5K*, *PLD*, and *DGK* genes affects the transport of nutrients by vesicles in the tapetum of male sterile wheat, thereby affecting male fertility. In this study, *PLD* was significantly upregulated (40-fold) in fertile anthers, while PA content was decreased. These significant gene expression differences suggest that *PLD* genes are involved in the regulation of fertility in rice PTGMS lines; however, whether *PLD* is involved in the male fertility process *via* the regulation of PA changes should be further investigated.

Finally, *4CL2* and *4CLL2*, which encode for 4-coumarate-CoA ligase, were obtained by enrichment in the phenylpropanoid metabolic pathway. First, 4CL converts p-coumaric acids to hydroxycinnamoyl-CoA. Subsequently, hydroxycinnamoyl-CoA is reduced to hydroxycinnamyl aldehyde and hydroxycinnamyl alcohol in the presence of cinnamoyl CoA reductase (*CCR*) and CAD to constitute various lignin monolignols (Schilmiller et al., 2009; Huang et al., 2010; Li et al., 2015). Zhang et al. (2018) found that deficiency of 4CL delayed the usage of phenylalanine in lignin synthesis and flavonoid biosynthesis, which causes male sterility in citrus. Other studies have suggested that the downregulation of 4CL leads to a decrease in the synthesis of the intermediate product feruloyl-CoA, which affects the synthesis of phenolic polymers and thus the synthesis of sporopollenin, inhibiting the pollen wall and pollen development processes leading to male sterility (Yang et al., 2020). In this study, we found that the *4CL1*, *4CL2*, *4CL3*, *4CL4*, and *4CL5* genes of the phenylpropanoid metabolic pathway are expressed at higher levels in fertile anthers than in sterile anthers (Figure 6C). As the first branching enzyme in the metabolic pathway of phenylpropane, high expression of *4CL* promoted the upregulation of *p*-coumaroyl-CoA, which might be responsible for the active flavonoid metabolism; in addition, the gene expression (*4CL, CCR, CAD*, and *COMT*) and metabolite contents (ferulic acid, coniferaldehyde and caffeyl alcohol) of the lignin G unit synthesis process were upregulated, while the average fold change of *4CL* (3.02) was at a maximum. Therefore, we consider the regulation of *4CL* genes in fertile anthers to be the main contributor to the process of exuberant flavonoid metabolism and pollen wall formation; in particular, the contribution of lignin G monolignol in the building of pollen walls and pollen might be due to *4CL*-specific effects. The specific contribution of *4CL* in the synthesis of G monomers is a focus of our future research.

## Conclusion

**Figure 8:**
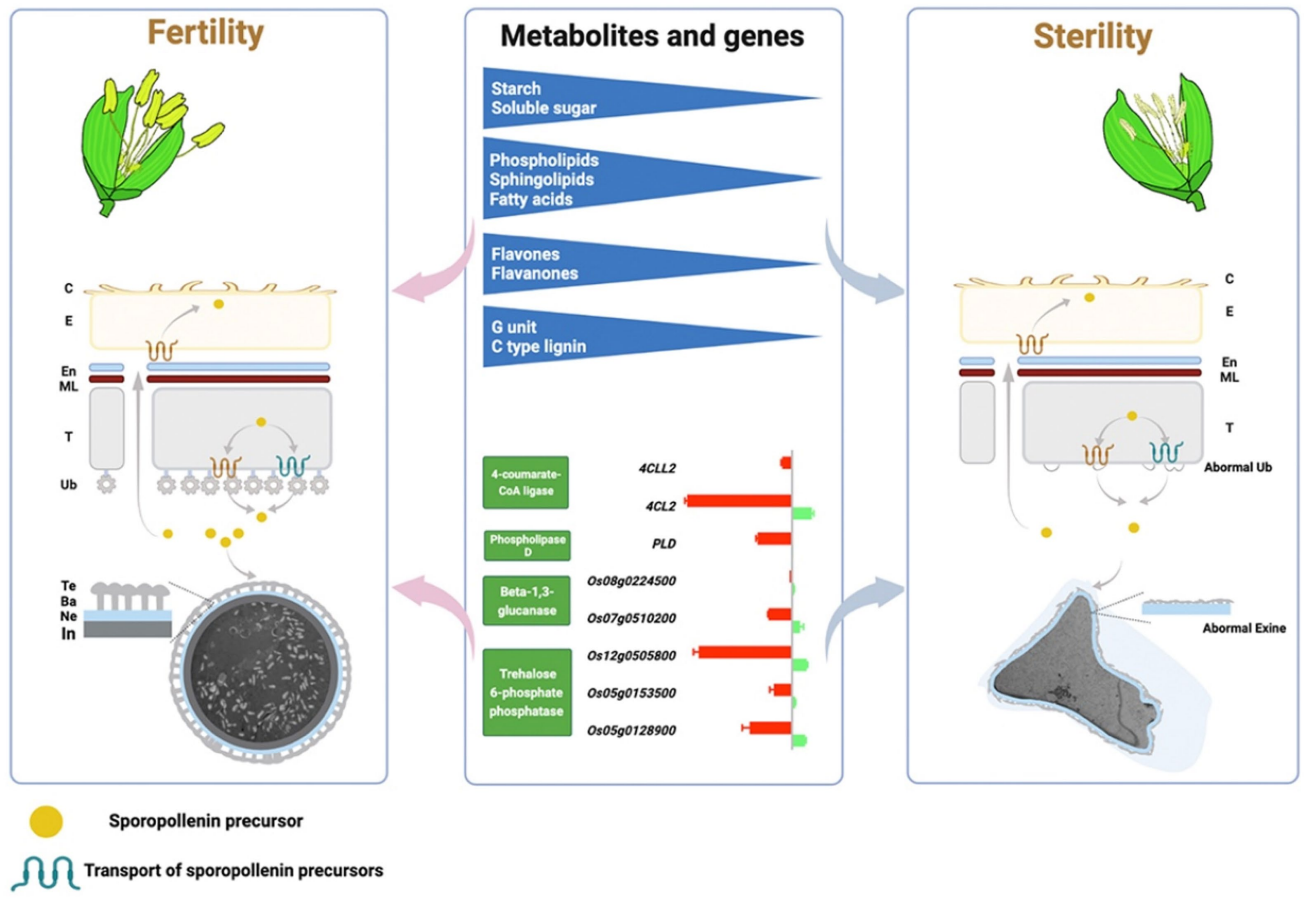
A model graph of the metabolites and related genes in the metabolic pathways of sugar, lipid and phenylpropanoid involved in pollen wall and male fertility formation. C, Anther cuticle; E, Epidermis; En, Endothecium; ML, Middle layer; T, Tapetum; Ub, Ubisch body; Te, Texine; Ba, Bacula; Ne, Nexine; In, Intin.

In this study, we performed a comprehensive analysis of comparative transcriptomic and targeted metabolomics in a group of NILs of rice PTGMS lines with different CTFTs, which illustrate the regulatory mechanisms of gene expression and metabolites in male fertility formation (Figure 8). First, the DEGs that respond to temperature changes and regulate male fertility were enriched in sugar, lipid and phenylpropanoid metabolic pathways in comparative transcriptomics. The sterile pollen grains showed no starch accumulation and abnormal pollen wall development in phenotypic analysis, an active sugar, lipid and flavonoid metabolic process was found in fertile anthers in targeted metabolomics, and lignin G monolignol and C-type lignin are major contributors to extrafloral wall formation. The critical genes in sugar metabolism (*TPP* and *beta-1,3-glucanase*), lipid metabolism (*PLD*) and phenylpropanoid metabolism (*4CL*) are essential for the formation of the pollen wall and male fertility. In conclusion, multiple metabolic processes cooperate to form male fertility, and sugar, lipid, and phenylpropanoid metabolic processes are essential.

## Data availability statement

The original contributions presented in the study are publicly available. This data can be found here: NCBI, PRJNA843872.

## Author contributions

HZ, YH, and YS contributed to conception and design of the study. YS organized the database and wrote the first draft of the manuscript. YS, MF, YA, LZ, and LW completed the experiment and performed the statistical analysis. HZ and YH provided experimental materials and financial support. All authors contributed to manuscript revision, read, and approved the submitted version.

## Funding

This work was supported by the Hubei Agricultural Science and Technology Innovation Center Program (2021-620-000-001-032) and the National Key R&D Program of China (2016YFD0300207).

## Conflict of interest

The authors declare that the research was conducted in the absence of any commercial or financial relationships that could be construed as a potential conflict of interest.

## Publisher’s note

All claims expressed in this article are solely those of the authors and do not necessarily represent those of their affiliated organizations, or those of the publisher, the editors and the reviewers. Any product that may be evaluated in this article, or claim that may be made by its manufacturer, is not guaranteed or endorsed by the publisher.
