# Supplementary material for: Combined analysis of transcriptome and metabolome reveals that sugar, lipid, and phenylpropane metabolism are essential for male fertility in temperature-induced male sterile rice
Source: Front Plant Sci. 2022 Jul 28;13:945105. doi: 10.3389/fpls.2022.945105 (PMC9370067; doi:10.3389/fpls.2022.945105)
Supplement: Supplementary file 1 [file Data_Sheet_1.docx]

Supplementary material

# Supplementary Figures


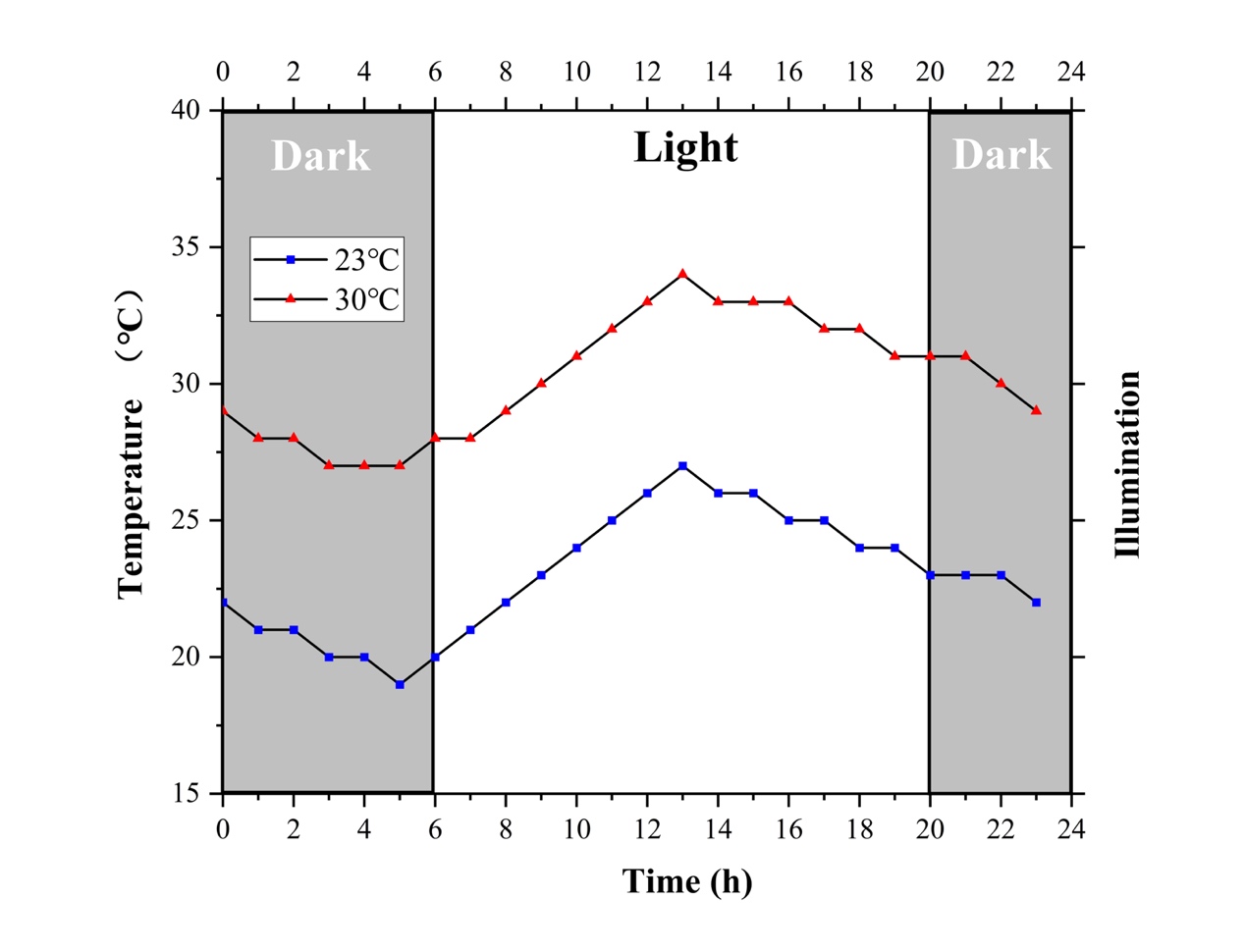


**Supplementary Figure 1.** Temperature setting conditions for the 23℃ and 30℃ treatments of P23, P28 and P28F. In the light intensity diagram, 14 hours of light, 10 hours of darkness.


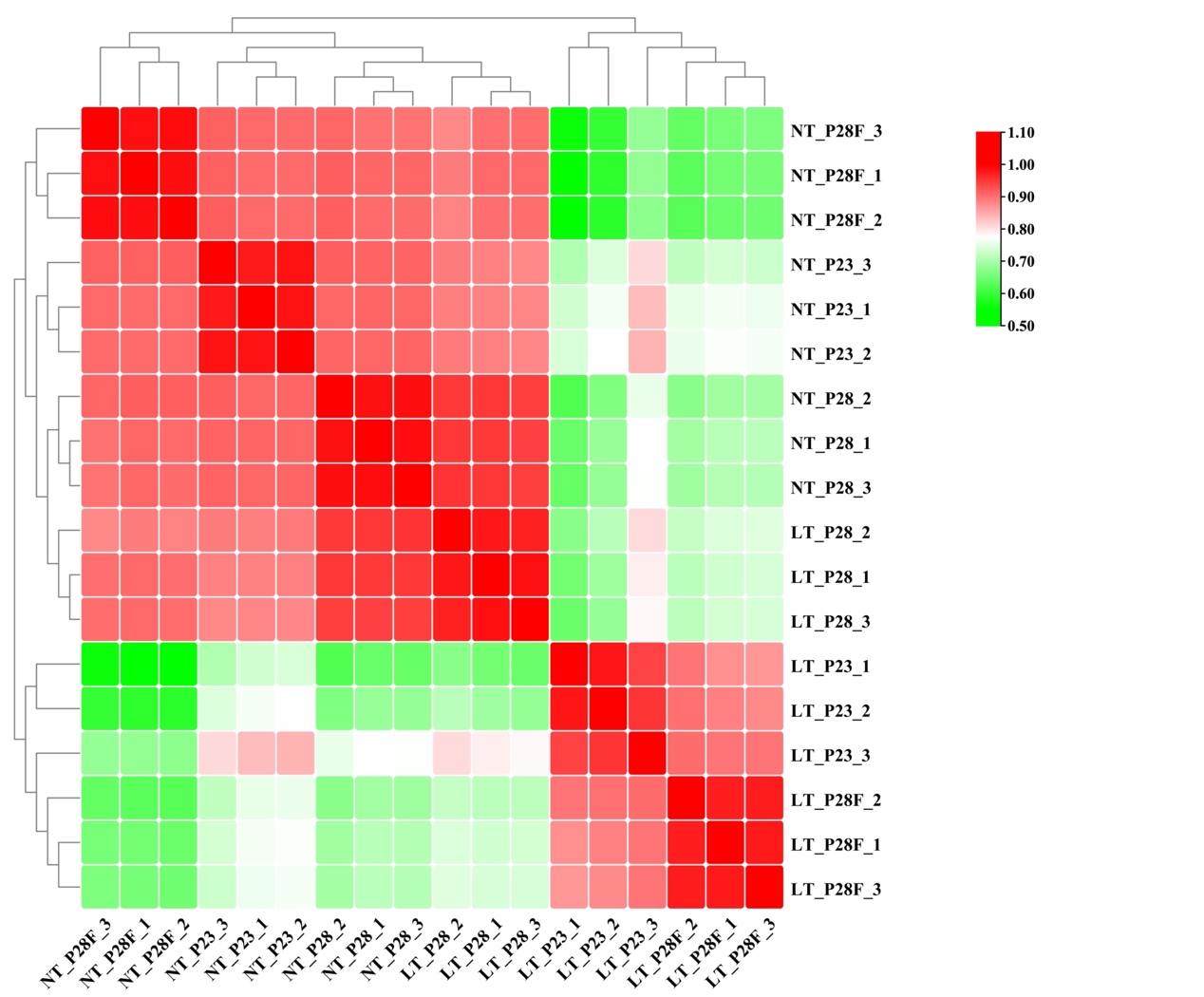


**Supplementary Figure 2.** Clustering heatmap between each sample by RNA sequencing.

###

**
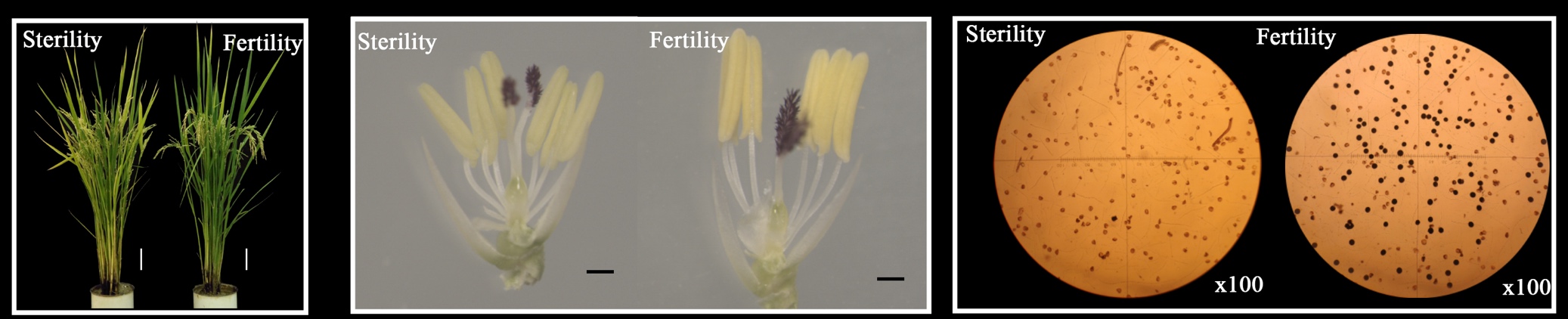
**

**Supplementary Figure 3.** Phenotype and pollen fertility of P23 plants treated with 21°C and 30°C.

**Supplementary Table 1.** Summary of sequencing and mapping results in P23, P28 and P28F under different temperature treatments.

| **Sample Sequencing** | | **Sequencing results** | | | **Mapping overview** | | | |
| --- | --- | --- | --- | --- | --- | --- | --- | --- |
|  |  | **Total reads** | **Raw bases** | **Q30 (%)** | **Cleaned reads** | **Reads mapped** | **Unique map** | **Mapping rate (%)** |
| LT_P28F_1 | 45779716 | 6.87G | 94.39 | 42791372 | 39876158 | 39192844 | 93.19 |  |
| LT_P28F_2 | 46383342 | 6.96G | 92.87 | 42629248 | 39228104 | 38482427 | 92.02 |  |
| LT_P28F_3 | 47775976 | 7.17G | 92.86 | 43703568 | 40156209 | 39448065 | 91.88 |  |
| LT_P28_1 | 47232850 | 7.08G | 94.63 | 44176662 | 41533996 | 40542733 | 94.02 |  |
| LT_P28_2 | 46642176 | 7.00G | 94.56 | 43416462 | 40836972 | 39827855 | 94.06 |  |
| LT_P28_3 | 46048102 | 6.91G | 94.63 | 42984266 | 40387167 | 39487312 | 93.96 |  |
| LT_P23_1 | 45229790 | 6.78G | 94.85 | 41729908 | 38858733 | 38214524 | 93.12 |  |
| LT_P23_2 | 47339552 | 7.10G | 94.09 | 42699300 | 39644460 | 38829364 | 92.85 |  |
| LT_P23_3 | 51972386 | 7.80G | 92.86 | 47436164 | 43600136 | 42818102 | 91.91 |  |
| NT_P28F_1 | 45230302 | 6.78G | 92.97 | 41578384 | 38495152 | 37460888 | 92.58 |  |
| NT_P28F_2 | 44458512 | 6.67G | 91.77 | 43701706 | 40584345 | 39585664 | 92.87 |  |
| NT_P28F_3 | 47324876 | 7.10G | 91.63 | 46392420 | 43015385 | 42005403 | 92.72 |  |
| NT_P28_1 | 45662674 | 6.85G | 91.42 | 44996124 | 41629757 | 40423600 | 92.52 |  |
| NT_P28_2 | 51573916 | 7.74G | 91.87 | 50321342 | 46816281 | 45646869 | 93.03 |  |
| NT_P28_3 | 45664362 | 6.85G | 92.00 | 44463370 | 41391549 | 40388410 | 93.09 |  |
| NT_P23_1 | 46672862 | 7.00G | 92.75 | 43194480 | 40655669 | 39561976 | 94.12 |  |
| NT_P23_2 | 44460730 | 6.67G | 93.25 | 40080534 | 37317852 | 36369978 | 93.11 |  |
| NT_P23_3 | 46341696 | 6.95G | 92.07 | 45193650 | 42072911 | 40953880 | 93.09 |  |

Q30: indicates the percentage of bases estimated to have base reliability of 99.9% or higher.

| **KEGGID** | **Description** | **pvalue** | **Count** |
| --- | --- | --- | --- |
| osa04016 | MAPK signaling pathway - plant | 1.54E-05 | 70 |
| osa00940 | Phenylpropanoid biosynthesis | 1.83E-05 | 93 |
| osa04075 | Plant hormone signal transduction | 2.58E-05 | 95 |
| osa00520 | Amino sugar and nucleotide sugar metabolism | 4.33E-04 | 62 |
| osa00592 | alpha-Linolenic acid metabolism | 1.92E-03 | 28 |
| osa04626 | Plant-pathogen interaction | 2.49E-03 | 82 |
| osa00052 | Galactose metabolism | 3.34E-03 | 26 |
| osa00500 | Starch and sucrose metabolism | 6.65E-03 | 68 |
| osa00944 | Flavone and flavonol biosynthesis | 9.46E-03 | 8 |
| osa00410 | beta-Alanine metabolism | 1.73E-02 | 19 |
| osa00941 | Flavonoid biosynthesis | 2.50E-02 | 16 |
| osa00480 | Glutathione metabolism | 2.63E-02 | 43 |
| osa00250 | Alanine, aspartate and glutamate metabolism | 2.82E-02 | 20 |
| osa00905 | Brassinosteroid biosynthesis | 3.15E-02 | 9 |
| osa00130 | Ubiquinone and other terpenoid-quinone biosynthesis | 3.80E-02 | 20 |
| osa00460 | Cyanoamino acid metabolism | 3.94E-02 | 23 |
| osa00650 | Butanoate metabolism | 6.49E-02 | 11 |
| osa00945 | Stilbenoid, diarylheptanoid and gingerol biosynthesis | 7.98E-02 | 8 |
| osa03440 | Homologous recombination | 8.85E-02 | 23 |
| osa00600 | Sphingolipid metabolism | 9.18E-02 | 11 |

**Supplementary Table 2.** Top 20 of the KEGG enrichment pathway for the other 1429 DEGs in P28.

**Supplementary Table 3.** The primer information for this study

| **Genes Name** | **Forward Primer** | **Reverse Primer** |
| --- | --- | --- |
| *PAL* | ACATCTACGGCGTCACCACC | GAAGATTCCGGCGTTGAGATG |
| *C4H* | CTCAAGTCCCAGCGCATGAA | CCACAGCGTCGTCTCGATAG |
| *4CL1* | TTCCACATCTACTCGCTCAAC | GCCTCAGTCATTCCATACCC |
| *4CL2* | ACATCTTCTCGCTCAACTCCG | GTCATCCCGTATCCCTGTCC |
| *4CL3* | GGAGACATCGGCTTCGTC | GGTGATTTCTGAGCCTTCTG |
| *4CL4* | ATCTACTCGCTCAACTCCGTG | CAGCCTCAGTCATCCCATAC |
| *4CL5* | GTCCCAATGAAGGACGATT | GAATTCCAGCAGCCAACTT |
| *CHS1* | ACTTCAGGATCACCAAGAGCG | CCCACTCCTTGATCGCCTTC |
| *CHS2* | ACCAACAGCGAGCACCTAAC | CCACTCTTTGATCGCCTCCT |
| *CHI* | GGATAGTTAGTTGCTCACCCGAT | TCCTCCAGGTACACGCCGAT |
| *F3H1* | GGAGGACAAGCTCCGGTTC | CCAGTCTTTCACCGCTTCAC |
| *F3H2* | GCTATCCTCTCCACCAGTTCG | GGCCGCTGAGGAATTTAGTG |
| *F3H3* | CAAAGCATGCCGTGATCTGG | GCCAGTATTGCAGCCAATCG |
| *FNS2* | TGCGGAAGAAGACAGACACA | CGGACCCGGCAGTAACAAC |
| *CCR1* | TGCAAGCAAACAGACAACCTGTA | TGGGGTAGGACTTCTTGGTG |
| *CAD2* | AGTACTGCAACAAGAGGATTTGG | GAAGTGCTTCAGTGGGCTGTA |
| *COMT* | CGCCCTCATGAACCAGGACA | GTACTCGAACGCCGTCATCC |
| *4CLL2* | AAGGCCATCCGCAAGTACAA | TTGAAGCCGCGAGTTCCTAC |
| *Os05g0128900* | GGTCATCTTGAAAGGGTGCTAT | TCCCGTTGTTCATCAATCTGT |
| *Os05g0153500* | GGATGCAGACCATGACTTCG | CTGGGTTTCTTGTCCACAGC |
| *Os12g0505800* | CACAGGGAGTTAGCAAGGGTC | AAATGCGGAGCTGGAGGT |
| *Os07g0510200* | CCTTCGCATTCGACAGCTA | GTGTTTGCCCCGTCAGATT |
| *Os08g0224500* | CCTCTACAAGGACCCCAACTT | TGAACTGTCGTGCGTACTGC |
| *Os03g0119100* | ATGCTGGCACGATGTCTACG | GTCTCCTCATCCCGTGTGC |
| *CYP704B2* | GGTTGAGATCGGGACGCTGT | ATCACCTTCTCTTGCTTGCCG |
| *OsTKPR1* | TTCTAAACCGAATCTTGTTGCG | TTATGAGGGCAGTTCGTTGATC |
| *OsC6* | GGGCTGTCGTCCATCGTGA | CGCAGGTGGAGATGATGTCC |
| *OsABCG15* | AGATGGCCCTCTCCCTATATTC | GGGTGTCATTGTAGGTGATCT |
| *OsSTRL2* | GACAACGTGCGACTGAGCA | CTTGAAGTAGAGCGTTCGCAG |
| *OsNP1* | GTCCATCTTCACCGATTCCG | CTTCACACCCATATACCTGCC |
| *Os02g0822400* | TGAGCCTTTACAAACCCAAGA | TCTCGCCTACATAGCCATCA |
| *Os01g0917500* | ATCAGTTCCAAGGTGACCGT | CATCTGCTCGGTTTCTCAGC |
| *Os02g0313400* | GCGTGCGAAATCCCCTTTAT | GCCTCTTCCACTCCCTCTAC |
| *Os07g0549600* | GGAGGAGTAACAAGGACCCA | ACCTCCAGCAGCGAGTCCCT |
| *Os02g0174100* | AAAGCTCCACTCAGCCATGT | AGGACACCGAAGAACATCCA |
| *Os02g0655200* | TGGTGGAACAGAAGAGGCATGG | GCATGAAGCAGAGAGTTGGCCTT |
| *Os02g0655200* | CCATCCCGAACTACACGTTC | GTAGGAGAAGACGCCATTGTAC |
| *Os09g0491532* | TCCTTTCATGAGCCAGGCAG | CCACTGGATTGAACCGGACA |

| **Years** | **Treatment** | **Pollen fertility** | **Seed setting rate (%)** |
| --- | --- | --- | --- |
| 2017 | 21℃-P23 | 42.67±6.96 | 34.87±1.97 |
|  | 21℃-P28 | 30.26±0.35 | 33.20±0.66 |
| 2018 | 21℃-P23 | 41.09±8.93 | 33.05±5.98 |
|  | 21℃-P28 | 32.20±0.50 | 33.33±0.34 |
| 2019 | 21℃-P23 | 43.72 ± 2.69 | 38.40 ± 7.87 |
|  | 21℃-P28 | 31.34±0.90 | 28.30±0.56 |
| 2020 | 21℃-P23 | 39.30±0.29 | 36.27 ± 5.99 |
|  | 21℃-P28 | 31.10±0.22 | 30.47 ± 1.22 |
| 2021 | 21℃-P23 | 42.30 ± 0.87 | 37.05 ± 3.29 |
|  | 21℃-P28 | 32.60±0.69 | 30.87 ± 2.21 |
| Mean (5 years) | 21℃-P23 | 41.82±3.94 | 35.93±5.02 |
|  | 21℃-P28 | 31.5±0.53 | 31.23±1.00 |

### **Supplementary Table 4.** Pollen fertility and seed‐setting rate of P23 and P28 under 2017-2021.

| Group | Abbreviations | Full name |
| --- | --- | --- |
| Phospholipids | PIP3 | Phosphatidylinositol triphosphate |
|  | PIP2 | Phosphatidylinositol diphosphate |
|  | PS | Phosphatidylserine |
|  | PI | Phosphatidylinositol |
|  | PG | Phosphatidylglycerol |
|  | PE | Phosphatidylethanolamine |
|  | PC | Phosphatidylcholine |
|  | PA | Phosphatidic acid |
|  | MLCL | Moon-lyso cardiolipin |
|  | LPS | Lyso phosphatidylserine |
|  | LPI | Lyso phosphatidylinositol |
|  | LPG | Lyso phosphatidylglycerol |
|  | LPE | Lyso phosphatidylethanolamine |
|  | LPC | Lyso phosphatidylcholine |
|  | LPA | Lysophosphatidic acid |
|  | DLCL | Di-lyso cardiolipin |
|  | CL | Cardiolipin |
| Sphingotpids | SPH | Sphingosine bases |
|  | SM | Sphingomyelin |
|  | GD2 | Ganglioside, disialo dihexosyl ceramide |
|  | GM3 | Ganglioside, monosialo dihexosyI ceramide |
|  | CerG2GNAc1 | Dihexosyl N-acetylhexosyl ceramide |
|  | CerG3GNAc1 | Trihexosyl N-acetylhexosyl ceramide |
|  | Hex1Cer | Hexosyl ceramide |
|  | Hex2Cer | Dihexosyl ceramide |
|  | Hex3Cer | Trihexosyl ceramide |
|  | Cer | Ceramide |
|  | CerP | Ceramide phosphate |
| Fatty acids and other lipids | WE | Wax esters (fatty acid esters) |
|  | SL | Semino lipid (galactosyl diglyceride sulfate) |
|  | PMe | Phosphatidylmethanol |
|  | PEt | Phosphatidylethanol |
|  | OAHFA | O-Acyl-(gamma-hydroxy) fatty acid |
|  | LPMe | Lyso phosphatidylmethanol |
|  | LPEt | Lyso phosphatidylethanol |
|  | FA | Fatty acid |
|  | cPA | Cyclic phosphatidic acid |
|  | Co | Coenzyme Q |
|  | AEA | N-Acylethanolamine |
|  | AcCa | Acyl Carnitine |

**Supplementary Table 5**. Abbreviated list

| Abbreviations | Full name |
| --- | --- |
| PTGMS | Photoperiod- and thermo-sensitive genic male sterility |
| CTFT | Critical temperature for fertility transformation |
| miRNA | MicroRNA |
| circRNA | Circular RNA |
| lncRNA | Long noncoding RNA |
| PCD | Premature programmed cell death |
| NILs | The near isogenic lines |
| cDNA | complementary DNA |
| FPKM | Fragments Per Kilobase of exon model per Million mapped fragments |
| UPLC | Ultra-Performance Liquid Chromatography |
| ESI | Electron spray ionization |
| MS | Mass spectrum |
| FC | Fold change |
| LT | Low temperature (23℃) |
| NT | Natural temperature (30℃) |
| PCD | Premature programmed cell death |
| NILs | The near isogenic lines |
| LD | Long day |
| GO | Gene ontology |
| KEGG | Kyoto Encyclopedia of Genes and Genomes |
| qRT-PCR | Quantitative real-time PCR |
| TEM | Transmission electron microscopy |
| SEM | Scanning electron microscopy |
| DEG | Differentially expressed gene |
| 4CL | 4-coumarate-coa ligase |
| ACOS | Acyl-coa synthetase |
| TAG | Triacylglycerol |
| *p*-CA | *p*-coumaric acid |
| *p*-BA | *p*-hydroxybenzoate |
| TPS | Trehalose-6-phosphate synthase |
| TPP | Trehalose-6-phosphate phosphatase |
| PLD | Phospholipase D |
| CCR | Cinnamoyl coa reductase |
| CAD | Cinnamyl alcohol dehydrogenase |
| RNAi | RNA interference |
